# Effect of Distance from Catalytic Synergy Group to Iron Porphyrin Center on Activity of G-Quadruplex/Hemin DNAzyme

**DOI:** 10.3390/molecules25153425

**Published:** 2020-07-28

**Authors:** Dehui Qiu, Jingang Mo, Yuan Liu, Jiangyan Zhang, Yongqiang Cheng, Xiaobo Zhang

**Affiliations:** 1 Key Laboratory of Medicinal Chemistry and Molecular Diagnosis, Ministry of Education, Key Laboratory of Analytical Science and Technology of Hebei Province, College of Chemistry and Environmental Science, Hebei University, Baoding 071002, China; Dehui.Qiu@outlook.com (D.Q.); liuzhuolan@live.com (Y.L.); hdzjy2005@163.com (J.Z.); 2 State Key Laboratory of Analytical Chemistry for Life Science, School of Chemistry & Chemical Engineering, Nanjing University, Nanjing 210023, China; 3 School of Life Science, Changchun Normal University, Changchun 130032, China; mojingang@ccsfu.edu.cn; 4 Institute of Life Science and Green Development, Hebei University, Baoding 071002, China

**Keywords:** G-quadruplex/Hemin, DNAzyme, catalytic activity, catalytic mechanism, second-cytosine, distance regulation

## Abstract

G-quadruplex/Hemin (G4/Hemin) complex has been widely used in biocatalysis and analytical applications. Meanwhile, compared with natural proteinous enzyme, its low catalytic activity is still limiting its applications. Even though several methods have been developed to enhance the peroxidation efficiency, the important core of the G4 design based enhancement mechanism is still indistinct. Here, we focus the mechanism study on the two most important microdomains: the iron porphyrin center and the catalytic synergy group within the 3′ flanking. These microdomains not only provide the pocket for the combination of substrate, but also offer the axial coordination for the accelerated formation of Compound I (catalytic intermediate). In order to obtain a more suitable space layout to further accelerate the catalytic process, we have used the bases within the 3′ flanking to precisely regulate the distance between microdomains. Finally, the position-dependent effect on catalytic enhancement is observed. When dC is positioned at the second-position of 3′ flanking, the newly obtained DNAzyme achieves an order of magnitude improvement compared to parent G4/Hemin in catalytic activity. The results highlight the influence of the distance between the catalytic synergy group and iron porphyrin center on the activity of DNAzyme, and provide insightful information for the design of highly active DNAzymes.

## 1. Introduction

G-quadruplex (G4) is a four-stranded nucleic acid structure formed by stacked guanine tetrads [1,2,3] and it can combine with Hemin (G4/Hemin) to mimic peroxidase for catalyzing redox reaction [4,5,6]. Compared with protein peroxidases, G4/Hemin possesses several significant advantages, including high cost-efficiency ratio, excellent adaptability to harsh conditions, and chemical modification potential [7,8,9]. As a promising DNAzyme, G4/Hemin DNAzyme can efficiently catalyze H_2_O_2_-mediated oxidation with several substrates including 2,2′-azino-bis(3-ethylbenzothiazoline-6-sulfonic acid) (ABTS) [5], 3,3′,5,5′-tetramethylbenzidine sulfate (TMB) [10], luminol [11], nicotinamide adenine dinucleotide (NADH) [12], and dopamine [13]. Therefore, G4/Hemin based flexible biosensing strategies have been widely applied to detect small biomolecules, nucleic acids, proteins in vitro and even in vivo [14,15,16,17,18,19,20]. Despite the listed advantages, the biocatalytic application of G4/Hemin has been limited due to its much lower activity than horseradish peroxidase (HRP) [21]. In order to enhance the activity of G4/Hemin, different methods have been proposed. For example, some exogenous catalytic synergy agents, such as adenosine triphosphate (ATP) [22,23], DOTA-templated synthetic G-quartet (DOTASQ) [24], nitrogenous buffers [6], and spermine [25], have been used as efficient additives to boost the catalytic activity of G4/Hemin. However, these exciting results only appear along with extremely high concentrations of above additives, which is very unfriendly for related applications, especially in vivo experiments.

**Figure 1:**
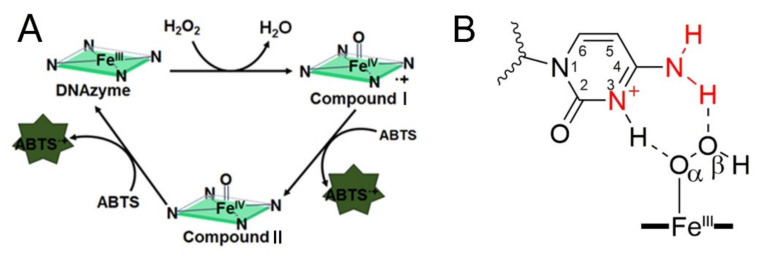
(**A**) Traditional peroxidation cycle facilitated by G-quadruplex DNAzyme for the ABTS-H_2_O_2_ reaction. (**B**) Proposed intermediate catalytic process, which is assisted with proximal flanking dC.

In recent years, several catalytic synergy group assisted G-quadruplex designs have been proposed to improve the catalytic activity. Using poly d(CCC), dA and poly dA as the flanking sequences, or changing the loop to poly dA and poly dC has been reported [26,27,28,29,30]. According to these reports, the G4-promoted oxidation of ABTS can be divided into three steps, which were presented in Figure 1A. Promoting the formation of Compound I is the important core of these activations. Figure 1B indicates the strategy that introducing proximal flanking catalytic synergy group (dA or dC) to accelerate the formation of the oxidation state of iron porphyrin (Compound I) [22]. However, it still lacks a systematic study on space layout regulation-based sequence optimization (the distance from catalytic synergy group to the iron porphyrin center). In this paper, different base combinations and distances are designed to screen highly active DNA enzymes. Finally, we found that when dC arranged on the second-position with first-position base is dT, dA or dC, the new obtained DNAzyme achieves an order of magnitude improvement in catalytic activity relative to the parent G4/Hemin system. Furthermore, our investigation also provides fundamental insights into how to design new-generation of DNAzyme with catalytic synergy group in optimal distance.

## 2. Results and Discussion

Previous study has indicated that the activation effect of the flanking sequences at the 3′ end was much higher than that at the 5′ end [27]. Therefore, we focus our study on the 3′ flanking sequences. Inspired by the coordination of His42 onto HRP center, both the unprotonated N and amino groups (-NH_2_) of the dA or dC have been considered as important candidates for improving the catalytic activity of DNAzyme (Supplementary Figure S1A,B, marked with red) [28]. So, the effect of distance from dA or dC to hemin center is investigated here for screening the DNAzymes with optimal catalytic activity. Considering the high stability of parallel G-quadruplex, G3T (5′-GGGTGGGTGGGTGGG-3′) was selected as the parent sequence. The 3′ flanking sequences are designed with different number of nucleotides, which contains dA (or dC) at the first to fourth positions (Supplementary Table S1). It should be noted that guanine (dG) was excluded at the first position of 3′ flanking to avoid the parent G4 structure (Supplementary Figure S2A) changed to non-parent G4 structure (Supplementary Figure S2B) [30]. Peroxidase-like activities of these synthesized G4/Hemin DNAzymes are characterized by measuring the initial rate (V_0_, nM/s) of the oxidation of ABTS.

Firstly, circular dichroism (CD) spectroscopy and isothermal difference spectra (IDS) are used to determine the stable parallel conformation of G4 structures in K^+^ solution. As shown in Supplementary Figure S3, the CD spectra of proposed G4 show positive peaks around 264 nm and negative peaks near 245 nm, which indicates the formation of parallel G-quadruplexes. Meanwhile, the addition of flanking nucleobases at 3′ end do not affect the formation of parallel G4. IDS in Supplementary Figure S4 for different number of flanking nucleobases also exhibit typical peaks of G4 with two positive peaks around 245 and 275 nm and a negative peak around 295 nm. These data indicate that the addition of 3′ flanking nucleobases does not affect the formation of G4.

**Figure 2:**
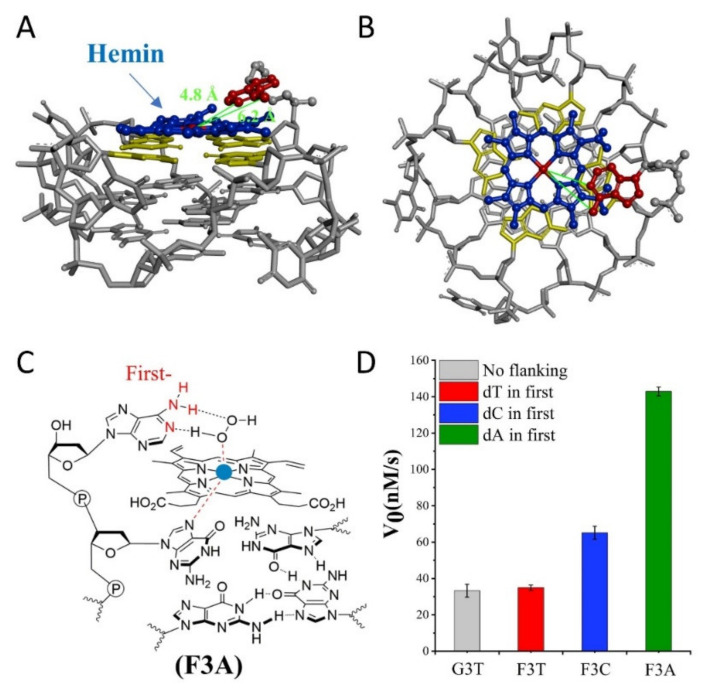
(**A**,**B**) Molecular model of F3A. A: side view, B: top view. Molecular docking studies were performed by PyMOL-2.3.1. with XRD crystal structure extracted from PDB:2LXV. (**C**) Schematic representation of hemin intermediate with F3A. The 3′ flanking dA makes the 6-NH_2_ and N1 group concertedly form two hydrogen bonds (N1-H-Oα and 6-NH-H-Oβ) with H_2_O_2_. (**D**) Summary of the catalytic activity of G3T and F3N.

Subsequently, we investigate the effect of distance from catalytic synergy group to iron porphyrin center on activity of G4/Hemin. Specifically, the dN is designed at the first position along the 3′ flanking of the parent G3T, and Figure 2D is their corresponding catalytic activity. Due to the inability to form a transition state similar to that shown in Figure 1B, the catalytic activity of F3T (35 ± 1 nM/s) is almost unchanged compared to that of G3T (33 ± 4 nM/s) (Figure 2D). This result experimentally proves that the presence of dT has little effect on the catalytic activity and can be used as an inert block for subsequent distance regulation. In contrast, the introduction of single dC or dA increases the catalytic activity of G4/Hemin system by nearly twice (F3C, 65 ± 4 nM/s) and four times (F3A, 143 ± 2 nM/s), respectively (Figure 2D). Although both bases have important synergistic catalytic groups, the nearly doubled difference in catalytic capacity of corresponding enzymes has led the research more interesting. Considering that there is only one added base, it is difficult for the synergistic catalytic group to participate in the formation of Compound I through the limited size of a single nucleotide. Because of this, under the same conditions, a larger dA molecular structure may shorten the distance between the synergistic catalytic group and the iron porphyrin center. As shown in Figure 2A,B, hemin stacks well upon the 3′ G-quartet of F3A. The hemin center is situated close to the N1 atom and 6-NH_2_ of dA in F3A with a distance about 4.8 Å and 6.2 Å. F3A provides a relatively suitable distance to make the 6-NH_2_ and N1 group concertedly form two hydrogen bonds (N1-H-Oα and 6-NH-H-Oβ) with H_2_O_2_ (Figure 2C). Therefore, finding a more suitable distance between the two will have the advantage of forming a better space layout to accelerate the catalytic process.

**Figure 3:**
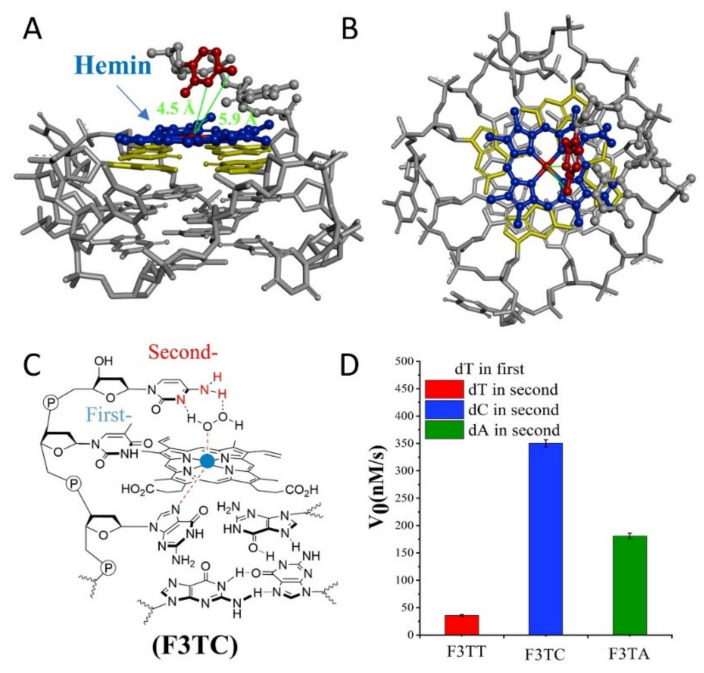
(**A**,**B**) Molecular model of F3TC. A: side view, B: top view. (**C**) Schematic representation of hemin intermediate with F3TC. The 3′ flanking dC makes the 4-NH_2_ and N3 group concertedly form two hydrogen bonds (N3-H-Oα and 4-NH-H-Oβ) with H_2_O_2_. (**D**) Summary of the catalytic activity of F3TN.

If the weaker performance of F3C is attributed to the distance, then the addition of another dT as first-position base between G-quartet and dN has been carried to solve this query (Figure 3). The catalytic capabilities of F3TT (32 ± 3 nM/s) and G3T (33 ± 4 nM/s) are very close, which once again proves the reliability of dT as a distance regulating element (Figure 2D and Figure 3D). But this increased first-dT, which converts F3C to F3TC, boosts the catalytic efficiency from 65 ± 4 nM/s (F3C) to 350 ± 7 nM/s (F3TC) (Figure 3C,D). As shown in Figure 3A,B, hemin stacks well upon the 3′-G-quartet of F3TC. The N3 atom and 4-NH_2_ of dC in F3TC are situated close to the hemin center with a distance about 4.5 Å and 5.9 Å. This indicated that the addition of first-dT can provide an appropriate distance for the coordination of dC with the substrate and porphyrin center, thus effectively enhancing the catalytic activity of G4/Hemin DNAzyme. But such a large increase in catalytic activity did not appear in the F3TA system (Figure 2D and Figure 3D). The limited activity enhancement from F3A (143 ± 2 nM/s) to F3TA (178 ± 4 nM/s) may indicate that the optimal distance between the catalytic synergy group and the catalytic center is near the second-position along the 3′ flanking. Therefore, one more added dT (F3TTN) has been introduced to determine the best result (Supplementary Figure S5). Compared with F3TC with dC placed in second-position, the catalytic activity of F3TTC decreased significantly (from 350 ± 7 nM/s to 78 ± 3 nM/s). In F3TTC system, the distances from the N3 atom and 4-NH_2_ of dC to hemin center enlarged to about 7.0 Å and 7.1 Å, which may exceed the distance required for H_2_O_2_ coordination (Supplementary Figure S5A,B). Base A with larger molecular size further increases the distance between the catalytic synergy group and the catalytic center, reducing the catalytic activity to 50 ± 1 nM/s (F3TTA). So far, the above experimental results based on dT for distance regulation show us a trend of position-dependent catalytic activity of G4/hemin DNAzyme system.

**Figure 4:**
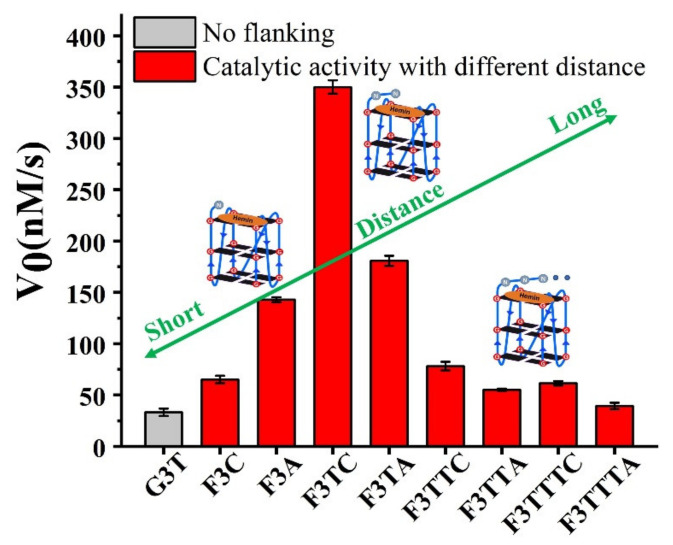
The effect of distance from catalytic synergy group (dC or dA) to iron porphyrin center on catalytic activity of G4/Hemin DNAzyme. Experiments were carried out in 10 mM Tris-HCl buffer (pH = 7.0, with 100 mM KCl, 0.05% Triton X-100, 1% DMSO,) at 25 °C with 0.4 μM G4, 0.6 mM H2O2, 0.6 mM ABTS and 0.8 μM hemin.

In order to better compare the activity of DNAzyme under different distance regulation, the related information of representative DNAzyme activity is summarized in Figure 4. When the flanking sequences compose of several dT, the catalytic activity will not be affected, since the dT lacks effective His42-like groups, unprotonated N and -NH_2_ (Supplementary Figure S6). While, dA or dC has unprotonated N and exocyclic amino group (-NH_2_) as catalytic synergy group, and dTs are arranged as the spacer to control the distance between dA (or dC) and hemin center. Comparing the structure of dC and dA nucleotides, the larger purine ring might provide enough distance for the coordination between proximal nucleobase with the iron porphyrin center. As a result, single dA can activate the DNAzyme, instead of single dC. And then, the optimum distance is acquired from F3TC (Figure 3C) with only one dT arranged as the spacer. In other words, the second-position along with 3′ flanking is the optimal. The best DNAzyme (F3TC) has achieved an order of magnitude improvement in catalytic activity (From 33 ± 4 nM/s to 350 ± 7 nM/s). Further addition of dTs will induce the decrease of catalytic activity, especially for dC. This position-dependent effect is also appropriate for dA: second-dA can also activate the DNAzyme best, even though the enhancement is not so obvious. In fact, all these distance adjustments are aimed to form the intermediate Compound 0 efficiently (Figure 1B and Figure 3B). F3TC provides a suitable distance to make the vicinal amino group and N3 group concertedly form two hydrogen bonds (N3-H-Oα and 4-NH-H-Oβ) with H_2_O_2_. N3 site of dC promotes the formation of Compound I by acting as a general acid-base catalyst and the 4-amino group of cytosine not only facilitates the binding of H_2_O_2_ to DNAzyme but also contributes to stabilizing the transition state for the Compound I formation by interacting with the β–oxygen atom. Since Compound I generation is the rate-limiting step in the peroxidase catalytic circle, the appropriate distance of dC accelerates the overall G4-DNAzyme catalytic circle via promoting Compound I formation (Figure 1A).

Although we have optimized the relevant distance through the dT interval, the actual DNAzyme design also needs to consider the possibility of co-existing catalytic synergy groups at other positions.

**Figure 5:**
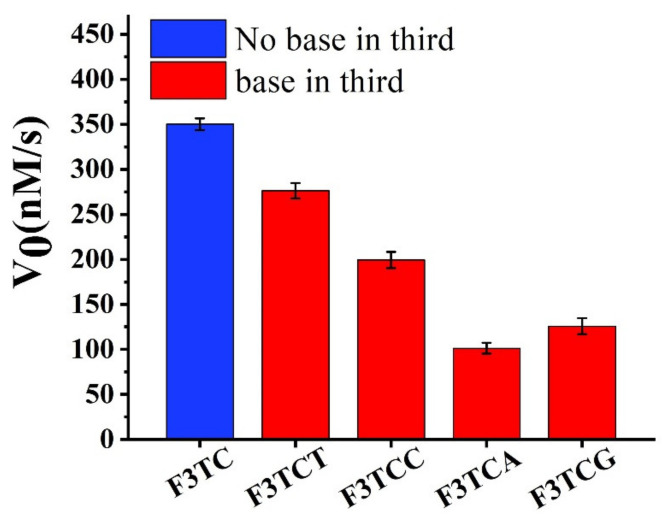
Catalytic performance of G-quadruplexes with F3TC and F3TCN (N = dT, dC, dA and dG).

Firstly, we consider extending a dN on the basis of F3TC for activity research (Figure 5). Compared to F3TC, the catalytic activities of F3TCN all decrease. Since dT does not interfere with the catalytic process, we believe that the decrease in the activity of F3TCT (277 ± 5 nM/s) comes from the increased steric effect. A greater degree of activity reduction from F3TCA (199 ± 4 nM/s), F3TCC (99 ± 2 nM/s) and F3TCG (125 ± 3 nM/s) may also involve the problem of newly introduced synergy catalytic groups to compete with the second-dC for substrate coordination. In addition, we have replaced the first nucleotide in the F3TC system (Supplementary Figure S7). Although the catalytic activity of F3CC (324 ± 2 nM/s) and F3AC (295 ± 2 nM/s) also decrease, the decline is relatively smaller than that in the F3TCN system. It can be considered that the group at the first nucleotide position of 3′ flanking has little effect on the catalytic system. Therefore, when designing DNAzymes, third-position related large steric hindrance and other groups that may interact with the substrate should be avoided.

Nucleotide extension along the flanking of the G4 sequences may cause the failure formation of stable G-quadruplexes conformation under different salt ion conditions. Hence, the other cations (sodium and ammonium) are also used for the formation of G-quadruplexes. Due to the design of our parent sequence, the type of cations will not influence the structure of G4s. From CD spectra (Supplementary Figures S3, S8, and S9) and IDS spectra (Supplementary Figures S4, S10, and S11), characteristic peaks indicated that the formation of parallel G4s would not affect by different cationic conditions, and the addition of flanking sequences as well. Not surprisingly, as shown in Supplementary Figure S12, DNAzymes have the same trend in catalytic activity under three cationic conditions, which also follows the rule of distance regulation. The catalytic activity of G4/Hemin buffered with NH_4_^+^ is higher than those of the same DNAzymes buffered with K^+^ or Na^+^, which is consistent with previously reported results: nitrogenous buffers appear to be preferable to the peroxidation than oxyanion buffers [5,28]. In addition, we studied the effect of pH on DNAzyme activity (Supplementary Figure S13), and found that neutral and weak acid conditions can maximize their effectiveness.

## 3. Materials and Methods

### 3.1. Materials and Reagents

PAGE or HPLC purified oligonucleotides were purchased from Sangon Biotech (Shanghai, China) without further purification. Dimethyl sulfoxide (DMSO), Hemin, 2,2′-azino-bis(3-ethylbenzothiazoline-6-sulfonic acid) (ABTS), sodium hydroxide (NaOH), hydrogen chloride (HCl), and tris(hydroxymethyl)aminomethane (Tris) were purchased from Sigma-Aldrich (Shanghai) Trading Co., Ltd. (Shanghai, China). Potassium chloride(KCl), sodium chloride (NaCl), ammonium chloride (NH_4_Cl), hydrogen peroxide (H_2_O_2_), and Triton X-100 were purchased from Aladin Ltd. (Shanghai, China). All other chemical reagents with analytical grade were used directly without further purification.

### 3.2. Preparation of DNA Solution

Oligonucleotides were dissolved in ultrapure water (18.2 MΩ·cm). The concentration of oligonucleotide solution was determined by UV absorbance at 260 nm using the molar extinction coefficients provided by IDT OligoAnalyzer 3.1 (http://sg.idtdna.com/calc/analyzer). The DNA solution was heated to 95 °C for 5 min, then cooled slowly to room temperature, and then stored at 4 °C overnight before use.

### 3.3. Preparation of Hemin and ABTS Solution

Hemin was dissolved in DMSO and diluted to 0.1 mM and then stored in the dark at 4 °C. Freshly prepared ABTS and H_2_O_2_ were dissolved in ultrapure water to 50 mM.

### 3.4. Preparation of G4/Hemin DNAzyme

The G4/Hemin DNAzyme was prepared by incubating G4 DNA (0.4 μM) with hemin (0.8 μM) in 10 mM Tris-HCl solution (pH = 7.0, 0.05% Triton X-100, 1% DMSO, 100 mM KCl (or NaCl/NH_4_Cl)) for 2 h at 25 °C.

### 3.5. Measurement of G4 DNAzyme Catalytic Activity (V_0_)

After the formation of G4/Hemin complexes, the substrate ABTS (0.6 mM) and H_2_O_2_ (0.6 mM) were added. Quartz cell path length was 1 cm. Catalytic activity measurement was followed by monitoring the absorbance of ABTS^+^ at 420 nm using a Cary100 (Agilent) spectrophotometer for 60 s using extinction coefficients of for ABTS·^+^ (36,000 M^−1^ cm^−1^ at 420 nm). The initial rate (V_0_, nM/s) of the oxidation reaction was obtained from the slope of the initial linear portion (the first 30 s) of the plot of absorbance versus reaction time. All kinetic measurements were repeated three times, and the background activity of hemin alone was subtracted.

### 3.6. Measurements of Circular Dichroism (CD)

CD spectra were collected on Chirascan circular dichroism spectropolarimeter (Applied Photophysics) in the 220–320 nm wavelength range. G4 solutions (2.5 μM strand concentration) were obtained by directly diluting the stock solutions of DNA (5 μM) into 10 mM Tris-HCl buffer (pH = 7.0, 100 mM KCl (or NaCl/NH_4_Cl)). The lamp was kept under a stable stream of dry purified nitrogen (99.999%) during experiments, and the measurements were repeated three times in 25 °C.

### 3.7. Measurements of Isothermal Difference Spectrum (IDS)

The UV spectrum was recorded for IDS measurement. G4 solutions (2.5 μM) were obtained by directly diluting the stock solutions of DNA (5 μM) into 10 mM Tris-HCl buffer (pH = 7.0, 100 mM KCl (or NaCl/NH_4_Cl)). The difference between the UV spectrum with the metal ion and the UV spectrum without metal ion is defined as the IDS and represents the spectral difference between the folded and the unfolded form.

## 4. Conclusions

In conclusion, all the results presented here show that the peroxidase-mimicking DNAzyme activity can be regulated by the 3′ end flanking sequences with a position-dependent effect. The appropriate position of dA/dC may accelerate the formation of Compound I and then enhance the catalytic activity of G4-based DNAzyme. Our results not only provide valuable insight regarding the important role of flanking sequences of G4s on their overall catalytic efficiency, beneficial to the subsequent design of highly active enzymes, but also pave the way for further improvements, to mimic natural proteinous enzymes. Furthermore, the catalytic activity position-dependent effect and distance regulation strategy found here can be further applied to potential applications.

## Supplementary Materials

The following are available online: Figure S1: The structure of adenine and cytosine nucleotide; Figure S2: The parent and possible non-parent G4 structure; Figures S3, S8, S9: CD spectra of G-quadruplexes; Figures S4, S10, S11: IDS of G-quadruplexes; Figure S5: Molecular model and schematic representation of hemin intermediate with F3TTC and summary of the catalytic activity of F3TTN; Figure S6: Catalytic performance of G-quadruplexes with several dT; Figure S7: Catalytic performance of G-quadruplexes with F3NC; Figure S12: Catalytic activity of G4/Hemin DNAzyme under different cation conditions; Figure S13: The effect of pH on catalytic activity of F3TC/Hemin DNAzyme; Table S1: The DNA sequences used in this work.
