# Supplementary material for: Effect of Distance from Catalytic Synergy Group to Iron Porphyrin Center on Activity of G-Quadruplex/Hemin DNAzyme
Source: Molecules. 2020 Jul 28;25(15):3425. doi: 10.3390/molecules25153425 (PMC7435396; doi:10.3390/molecules25153425)
Supplement: Supplementary file 1 [file molecules-25-03425-s001.pdf]

Supplementary Materials

Effect of Distance from Catalytic Synergy Group to Iron Porphyrin Center on Activity of G-Quadruplex/Hemin DNAzyme

Dehui Qiu [1,2,†], Jingang Mo [3,†], Yuan Liu [1], Jiangyan Zhang [1], Yongqiang Cheng [1,4,*] and Xiaobo Zhang [2,*]

[1] Key Laboratory of Medicinal Chemistry and Molecular Diagnosis, Ministry of Education, Key Laboratory of Analytical Science and Technology of Hebei Province, College of Chemistry and Environmental Science, Hebei University, Baoding 071002, P.R. China; Dehui.Qiu@outlook.com (D.Q.); liuzhuolan@live.com (Y.L.); hdzjy2005@163.com (J.Z.)

[2] State Key Laboratory of Analytical Chemistry for Life Science, School of Chemistry & Chemical Engineering, Nanjing University, Nanjing, 210023, China

[3] School of Life Science, Changchun Normal University, Changchun 130032, China; mojingang@ccsfu.edu.cn (J.M.)

[4] Institute of Life Science and Green Development, Hebei University, Baoding 071002, China

* Correspondence: yqcheng@hbu.edu.cn (Y.C.); zhangxiaobo@snnu.edu.cn (X.Z.)

† These authors contribute equally to this work.

Table S1. The DNA sequences used in this work

Name	Sequences (5´–3´)
**G3T**	GGGTGGGTGGGTGGG
**F3A**	GGGTGGGTGGGTGGGA
**F3C**	GGGTGGGTGGGTGGGC
**F3T**	GGGTGGGTGGGTGGGT
**F3CA**	GGGTGGGTGGGTGGGCA
**F3CC**	GGGTGGGTGGGTGGGCC
**F3TA**	GGGTGGGTGGGTGGGTA
**F3TC**	GGGTGGGTGGGTGGGTC
**F3TT**	GGGTGGGTGGGTGGGTT
**F3TTT**	GGGTGGGTGGGTGGGTTT
**F3TTC**	GGGTGGGTGGGTGGGTTC
**F3TTA**	GGGTGGGTGGGTGGGTTA
**F3TCT**	GGGTGGGTGGGTGGGTCT
**F3TCC**	GGGTGGGTGGGTGGGTCC
**F3TCA**	GGGTGGGTGGGTGGGTCA
**F3TCG**	GGGTGGGTGGGTGGGTCG
**F3TTTT**	GGGTGGGTGGGTGGGTTTT
**F3TTTC**	GGGTGGGTGGGTGGGTTTC
**F3TTTA**	GGGTGGGTGGGTGGGTTTA

SUPPLEMENTARY FIGURES

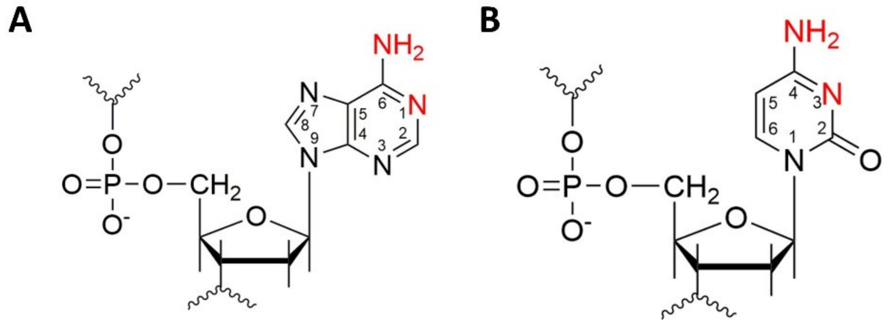

Fig. S1. (A) The structure of adenine nucleotide. (B) The structure of cytosine nucleotide. The atoms marked in red are coordination points of catalytic synergy group for improving the catalytic activity.

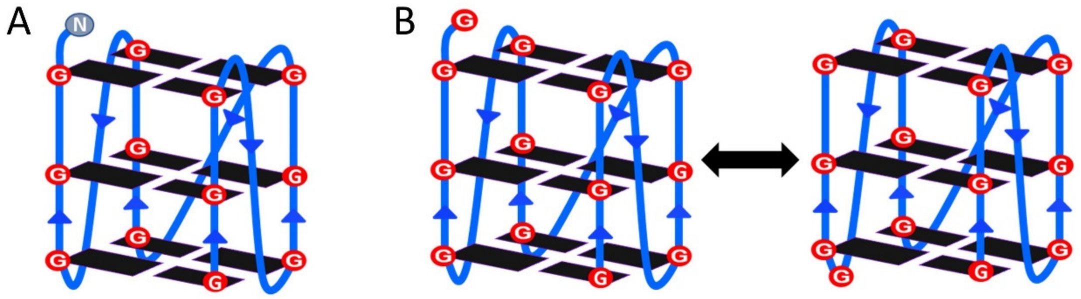

Fig. S2. (A) The parent G4 structure of F3N. (B) The added 3′ flanking dG may cause non-parent G4 structure [1].

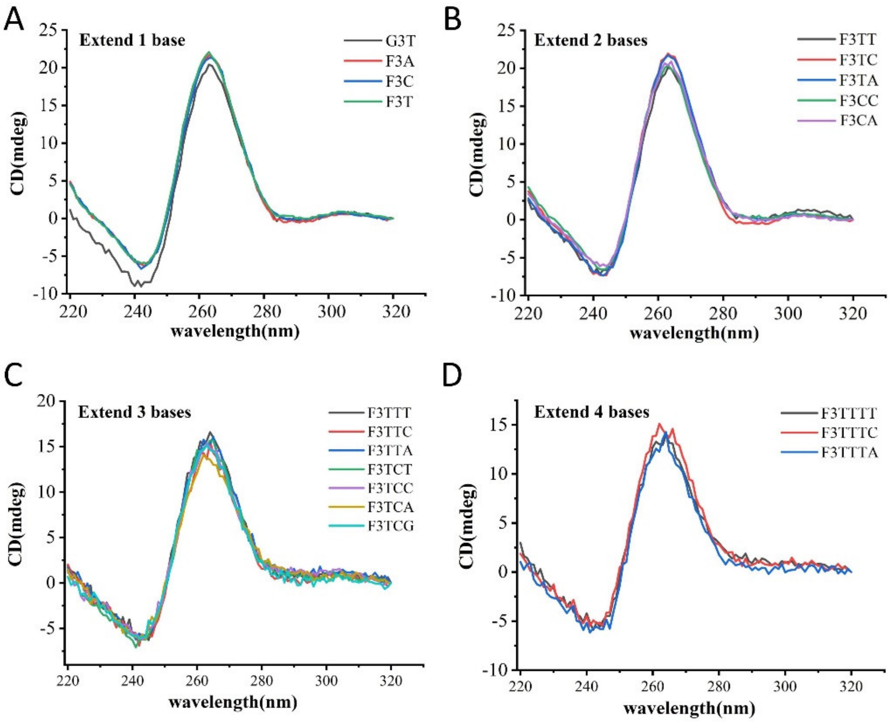

Fig. S3. CD spectra of the parallel G-quadruplexes extended with different bases. These DNA samples (2.5 μM) were stabilized in 10 mM Tris-HCl buffer (pH=7.0, 100 mM KCl).

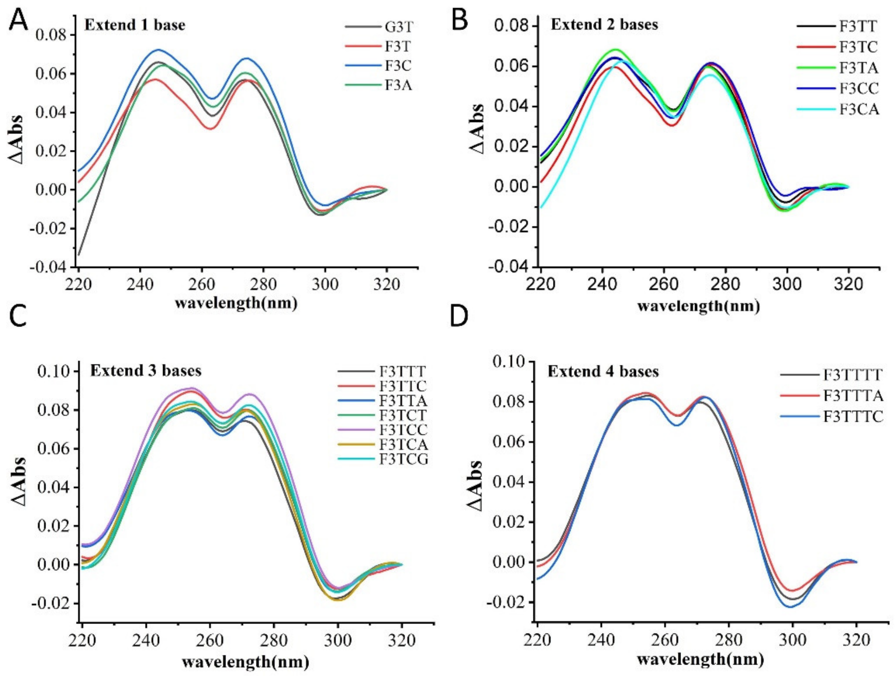

Fig. S4. IDS of the parallel G-quadruplexes extended with different bases. These DNA samples (2.5 μM) were stabilized in 10 mM Tris-HCl buffer (pH=7.0, 100 mM KCl).

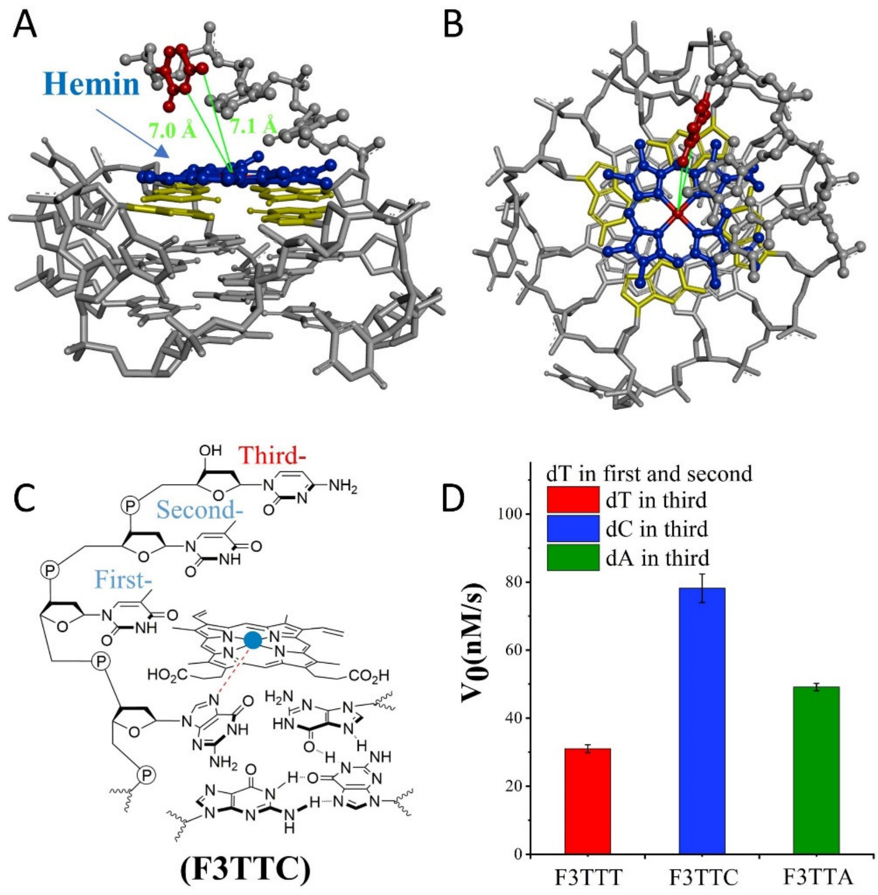

Fig. S5. (A, B) Molecular model of F3TTC. A: side view, B: top view. (C) Schematic representation of hemin intermediate with F3TTC. (D) Summary of the catalytic activity of F3TTN.

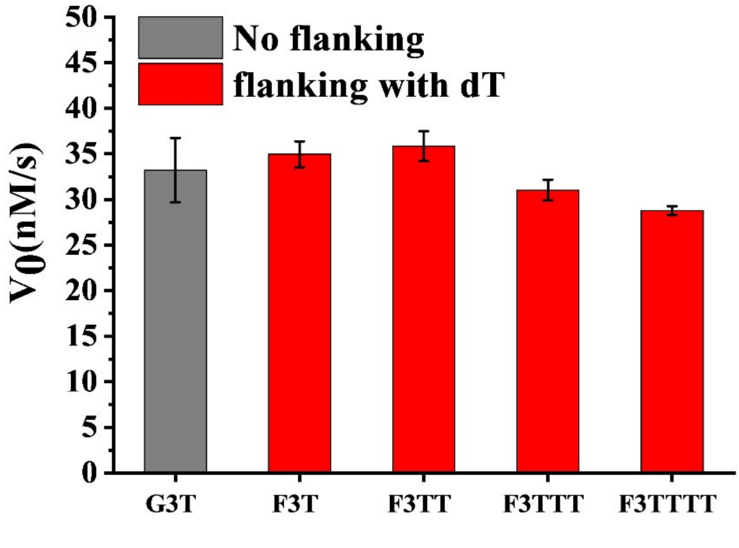

Fig. S6. Catalytic performance of G-quadruplexes with several dT.

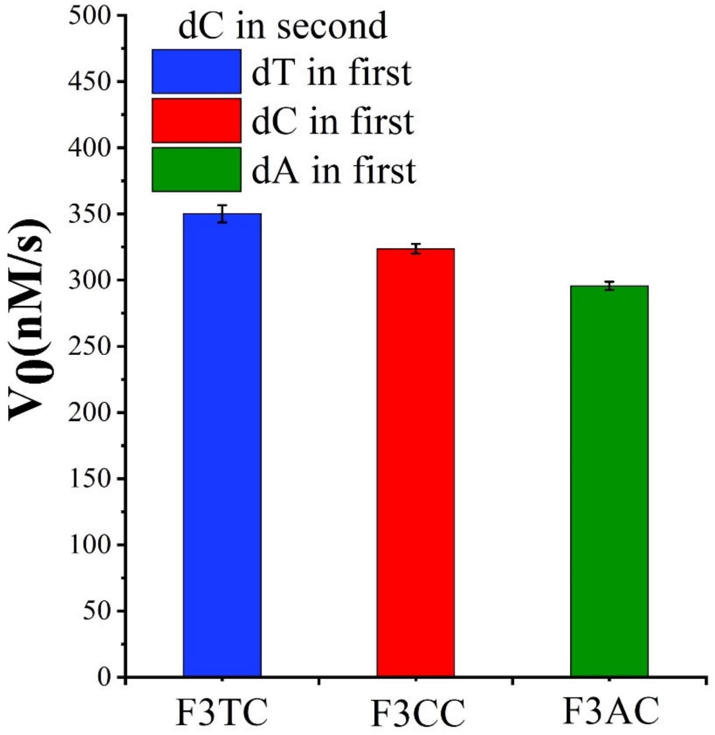

Fig. S7. Catalytic performance of G-quadruplexes with F3NC (N=dT, dC, and dA).

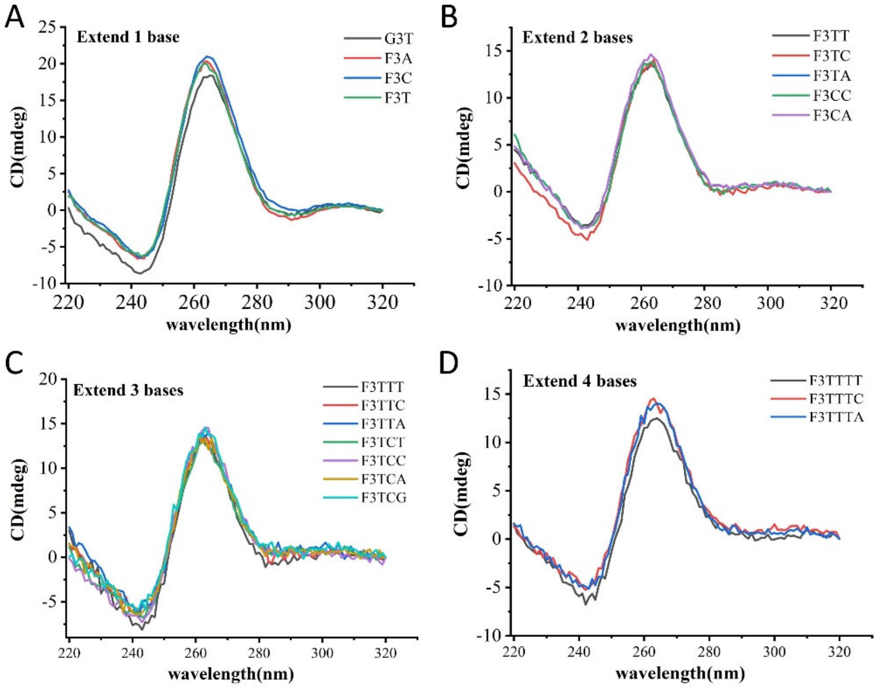

Fig. S8. CD spectra of the parallel G-quadruplexes extended with different bases. These DNA samples (2.5 μM) were stabilized in 10 mM Tris-HCl buffer (pH=7.0, 100 mM NaCl).

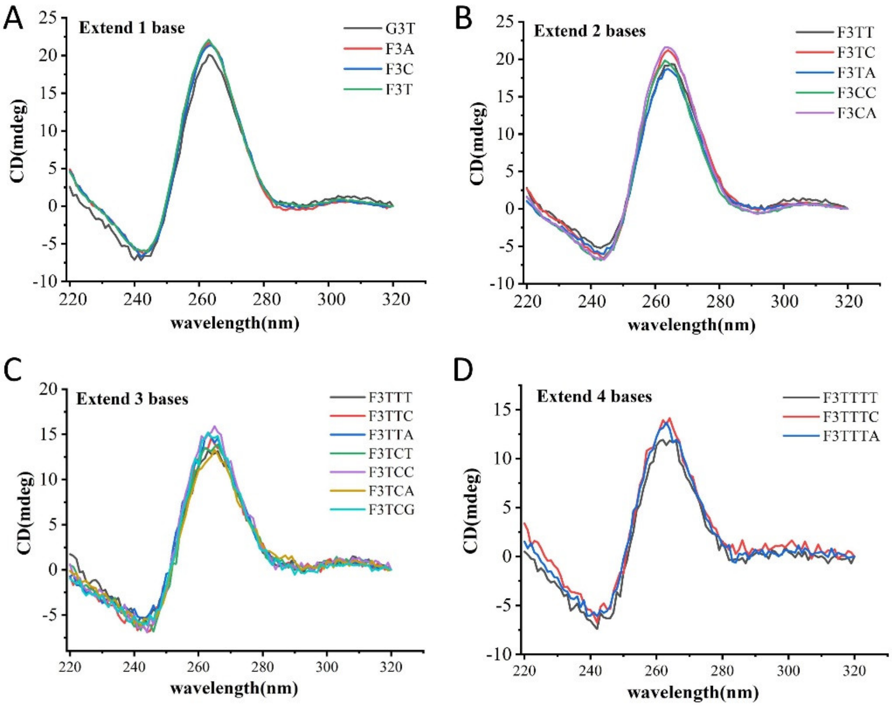

Fig. S9. CD spectra of the parallel G-quadruplexes extended with different bases. These DNA samples (2.5 μM) were stabilized in 10 mM Tris-HCl buffer (pH=7.0, 100 mM NH_4Cl).

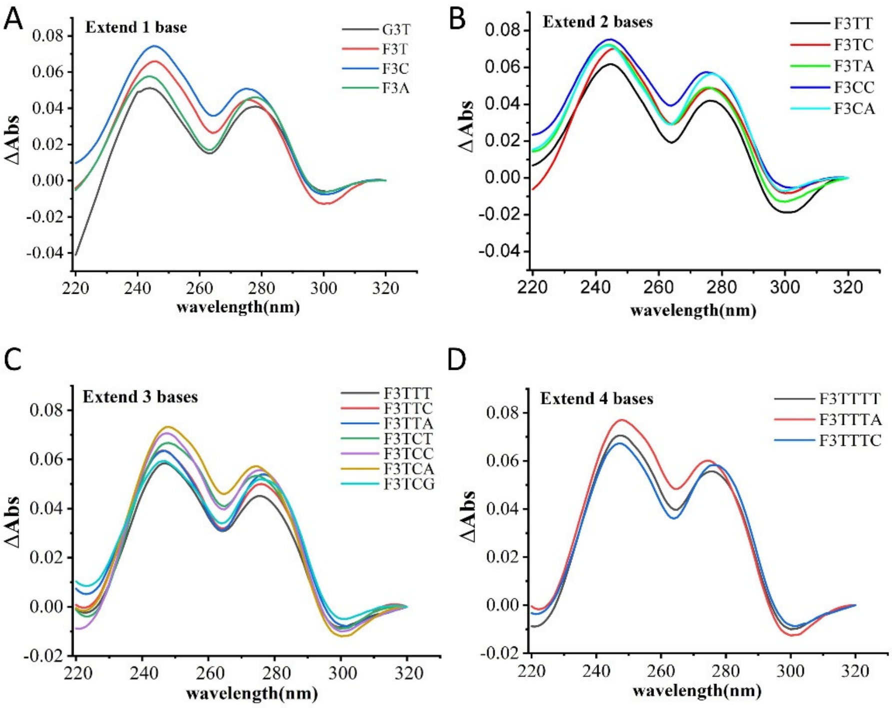

Fig. S10. IDS of the parallel G-quadruplexes extended with different bases. These DNA samples (2.5 μM) were stabilized in 10 mM Tris-HCl buffer (pH=7.0, 100 mM NaCl).

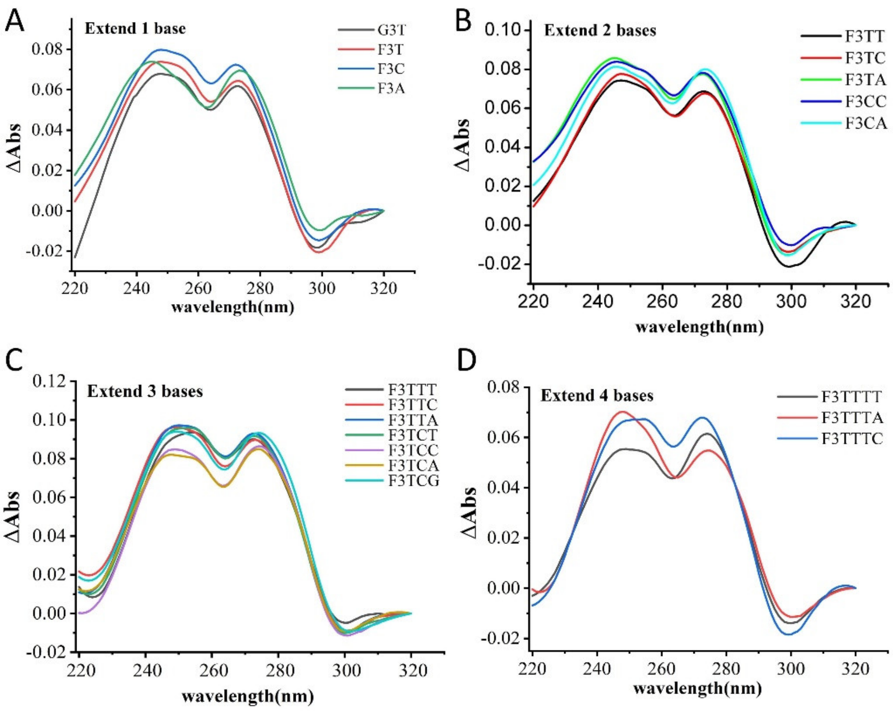

Fig. S11. IDS of the parallel G-quadruplexes extended with different bases. These DNA samples (2.5 μM) were stabilized in 10 mM Tris-HCl buffer (pH=7.0, 100 mM NH_4Cl).

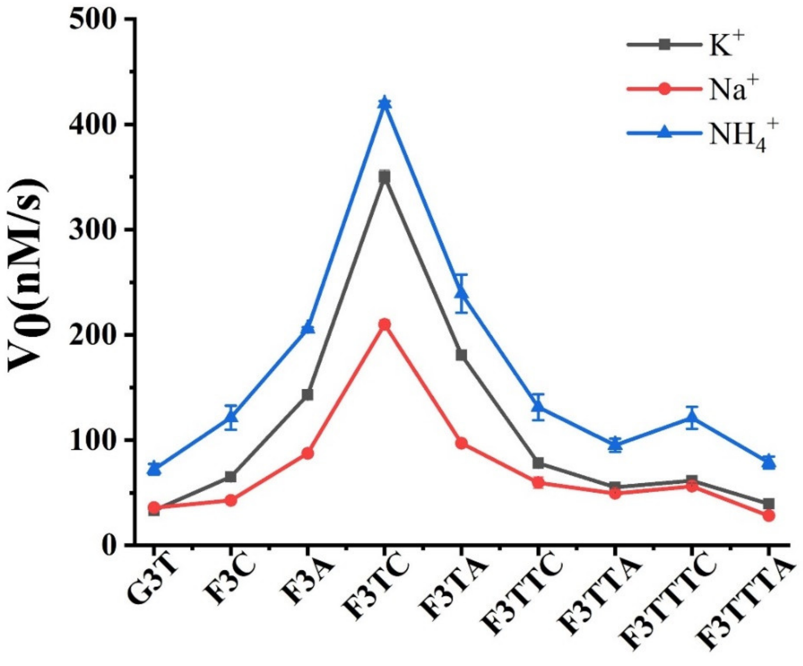

Fig. S12. The effect of distance from catalytic synergy group (dC or dA) to iron porphyrin center, under different cations (Na^+, K^+, NH_4^+), on catalytic activity of G4/Hemin DNAzyme. Experiments were carried out in 10 mM Tris-HCl buffer (pH=7.0, with 100 mM different cations, 0.05% Triton X-100, 1% DMSO,) at 25 °C with 0.4 μM G4, 0.6 mM H_2O_2, 0.6 mM ABTS and 0.8 μM hemin.

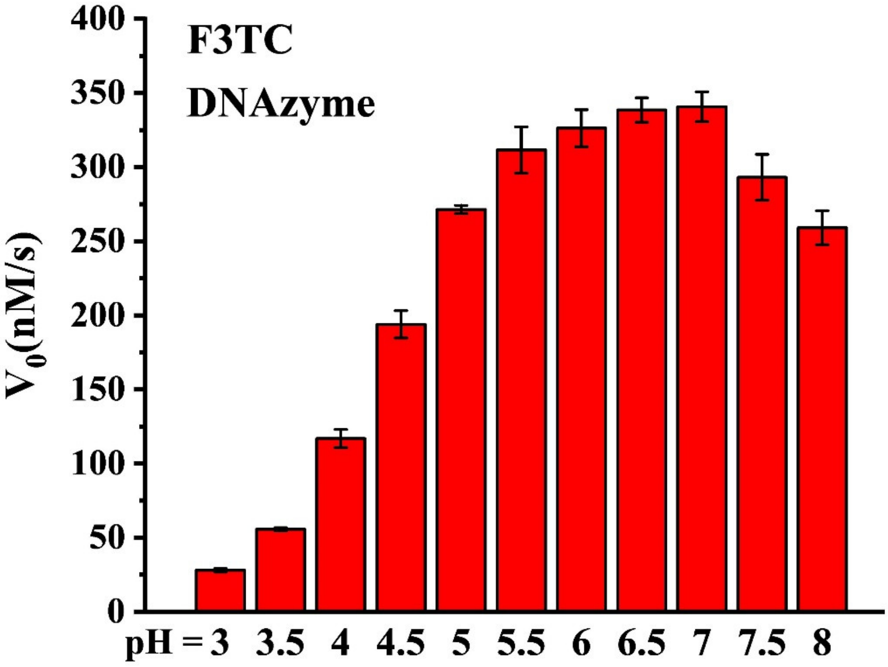

Fig. S13. The effect of pH on catalytic activity of F3TC/Hemin DNAzyme. Experiments were carried out in 10 mM B-R buffer (pH=3.0-8.0, with 100 mM K^+, 0.05% Triton X-100, 1% DMSO,) at 25 °C with 0.4 μM G4, 0.6 mM H_2O_2, 0.6 mM ABTS and 0.8 μM hemin.

REFERENCES

[1] W. Li, S. Chen, D. Xu, et al., Chem.-Eur. J. 24 (2018) 14500-14505.
